# The Unfinished Story of Sickle Cell Nephropathy: A Narrative Review of Knowledge Gaps and Research Imperatives

**DOI:** 10.3390/jcm15187185

**Published:** 2026-09-16

**Authors:** Mohammed Somaili

**Affiliations:** Department of Medicine, College of Medicine, Jazan University, Jazan 45142, Saudi Arabia; misomaili@jazanu.edu.sa; Tel.: +96-6557686546

**Keywords:** sickle cell nephropathy, chronic kidney disease, renal biomarkers, genetic risk, renoprotective therapies

## Abstract

Sickle cell nephropathy (SCN) is one of the most consequential yet under-recognized complications of sickle cell disease (SCD), contributing significantly to long-term morbidity and premature mortality. Despite its clinical importance, SCN often escapes early detection, in part because standard measures of kidney function can mask disease that is already well established. This narrative review synthesizes current evidence on SCN, examining the diagnostic strategies and the evolving therapeutic landscape—with particular attention paid to hydroxyurea, erythropoiesis-stimulating agents, and renal replacement therapy—and identifies the research gaps that must be addressed to improve outcomes for this patient population.

## 1. Introduction

Sickle cell disease (SCD) comprises a group of inherited hemoglobinopathies defined by the presence of hemoglobin S (HbS), which polymerizes under conditions such as infection, dehydration, and hypoxia [1]. This polymerization drives red blood cell sickling and recurrent vaso-occlusive events across multiple organ systems, with the kidney being among the most vulnerable organs to cumulative damage [2].

Sickle cell nephropathy (SCN) is a common complication of SCD, although its reported prevalence varies substantially according to age, SCD genotype, population, and the definition of kidney involvement used. Renal abnormalities are particularly frequent in sickle cell anemia and represent an important component of the overall disease burden [3].

SCN encompasses a broad clinical spectrum, ranging from impaired urinary concentrating ability and glomerular hyperfiltration to albuminuria, proteinuria, CKD, and eventually kidney failure [4]. Renal abnormalities may develop early in life and initially remain clinically silent. In a recent multicenter natural-history study of adults with sickle cell anemia, persistent albuminuria was present in 35.7% of participants, highlighting albuminuria as an important manifestation of SCN [4,5].

The natural history of SCN is incompletely characterized but it appears to involve a progressive sequence of renal abnormalities in a subset of patients, beginning with hyperfiltration and tubular dysfunction, followed by albuminuria and progressive decline in kidney function, with some patients ultimately developing kidney failure. However, progression is heterogeneous, and the factors determining which patients develop progressive CKD remain poorly understood [6,7].

SCN is also an important determinant of prognosis in SCD. The development of CKD and kidney failure is associated with substantially increased morbidity and mortality, making kidney involvement an important contributor to the long-term outcomes of patients with SCD [5].

Despite increasing recognition of SCN, its diagnosis and management remain challenging [8]. Conventional markers such as serum creatinine, estimated glomerular filtration rate (eGFR), and albuminuria may not accurately reflect early kidney injury in SCN. In addition, several primary and secondary glomerular diseases can mimic SCN, making the diagnosis particularly difficult when kidney function declines or proteinuria is disproportionate. At the same time, evidence supporting many kidney-specific therapies remains limited, while new disease-modifying and renoprotective treatments are emerging [9,10].

Most previous studies focused on individual aspects of SCN, including its pathophysiology, diagnosis, or treatment [11]. However, important gaps remain in integrating these areas into a practical clinical approach, particularly across the progression from early kidney involvement to ESKD [12]. This review therefore provides a clinically focused overview of SCN, bringing together the current challenges in diagnosis and differential diagnosis, available disease-modifying and renoprotective therapies, management of dialysis-dependent kidney failure, and the role of kidney transplantation and curative therapies such as hematopoietic stem cell transplantation and gene therapy. I also highlight the major evidence gaps that should guide future research.

## 2. Methods

### 2.1. Search Strategy and Information Sources

A comprehensive literature search was conducted across multiple electronic databases, including PubMed/MEDLINE, Embase, Scopus, and the Cochrane Library. The search was designed to identify peer-reviewed articles published from 2000 to the present as the principal search period, with emphasis on diagnostic challenges and treatment options—including renal replacement therapy—in sickle cell nephropathy. The search strategy combined Medical Subject Headings (MeSH) terms with free-text keywords. Boolean operators (AND, OR) were used to refine the results. Search terms were grouped into three categories: population-level terms (“sickle cell disease” and “sickle cell nephropathy”); condition-specific terms (“albuminuria,” “proteinuria,” “chronic kidney disease,” and “end-stage renal disease”); and terms related to management and diagnostics (“diagnostic challenges,” “biomarkers,” “creatinine clearance,” “hydroxyurea,” “ACE inhibitors,” “SGLT2 inhibitors,” “erythropoiesis-stimulating agents,” “stem cell transplantation,” “dialysis,” and “kidney transplantation”) [13].

### 2.2. Inclusion and Exclusion Criteria

To ensure the relevance and quality of the evidence presented, specific criteria were established for article selection (Table 1):

### 2.3. Study Selection and Data Synthesis

Study selection followed a multi-stage process. Titles and abstracts were first screened for relevance to the review’s core objectives—diagnostic challenges and management options—after which full-text versions of potentially eligible articles were retrieved and assessed against the inclusion criteria. The reference lists of retrieved articles and relevant review papers were also manually searched using a “snowballing” technique to capture additional landmark studies, including relevant publications predating the principal search period, that were not identified through the primary search. Findings were synthesized narratively and organized into three thematic blocks:

- Pathophysiological basis—linking hemoglobin S polymerization to renal injury.
- Diagnostic dilemmas—addressing the limitations of conventional markers, such as serum creatinine, in the SCN population.
- Therapeutic landscape—evaluating current and future pharmacological management strategies.

### 2.4. Quality Assessment

As this was a narrative rather than a systematic review, a formal risk-of-bias assessment using a single standardized instrument was not performed. Instead, the strength and limitations of the available evidence were evaluated according to study design and methodological characteristics, including sample size, prospective versus retrospective design, duration of follow-up, consistency of findings, clinical relevance, and applicability to SCN. Priority was given to systematic reviews and meta-analyses, randomized controlled trials, large prospective cohort studies, and evidence-based clinical practice guidelines where available. Lower-level evidence, including retrospective studies, small observational cohorts, case series, and expert opinion, was incorporated when higher-quality evidence was limited, particularly in areas where SCN-specific data remain sparse. The limitations and uncertainty associated with these data are acknowledged throughout the review, and conclusions are framed according to the strength and consistency of the available evidence [14].

## 3. Results

### 3.1. Pathophysiological Basis

Evolving paradigms and conceptual frameworks: the multi-hit hypothesis.

Understanding of SCN has evolved considerably, and current conceptual models now inform both research priorities and clinical practice [15]. Early descriptions of renal involvement in SCD focused largely on isolated hemodynamic changes; this view has since given way to a multi-hit hypothesis, in which hemolysis, chronic inflammation, oxidative stress, and genetic modifiers act in concert to drive injury. Together, these mechanisms produce progressive glomerular, tubular, vascular, and interstitial damage that culminates in chronic kidney disease (CKD) and, in a subset of patients, in end-stage kidney disease (ESKD) [16]. The individual components of this multi-hit model are discussed in more detail below.

#### 3.1.1. First Hit: Medullary Sickling and Microvascular Occlusion

The earliest pathogenic event in SCN is recurrent sickling within the vasa recta, driven by a combination of HbS deoxygenation, increased rigidity of sickled erythrocytes, and the inherently slow blood flow through these narrow vessels. Together, these factors promote vascular obstruction and local ischemia. Repeated cycles of vaso-occlusion lead to microinfarction, ischemia–reperfusion injury, capillary rarefaction, and progressive loss of medullary architecture [17]. Clinically, this manifests as hyposthenuria (impaired urinary concentrating ability), polyuria, nocturia, and early tubular dysfunction—findings that can appear as early as childhood before overt CKD becomes apparent [11,18].

#### 3.1.2. Second Hit: Chronic Hemolysis and Endothelial Dysfunction

Sickle cell disease is also marked by chronic intravascular hemolysis, which releases free hemoglobin, free heme, and iron-containing oxidative species into the circulation. These byproducts avidly scavenge nitric oxide (NO), driving the reaction:

*Hb* + *NO* → *MetHb* + *NO*_3^−^_

The resulting nitric oxide depletion leads to functional vasoconstriction, endothelial dysfunction, reduced renal perfusion, and increased vascular stiffness [3,17]. In response, the damaged endothelium upregulates adhesion molecules—including VCAM-1, ICAM-1, and selectins—which promote increased adhesion of sickled erythrocytes, leukocytes, and platelets, further amplifying vaso-occlusion [15].

#### 3.1.3. Third Hit: Glomerular Hyperfiltration

Glomerular hyperfiltration is among the earliest detectable renal abnormalities in SCD. Loss of medullary blood flow stimulates prostaglandin release, causing afferent arteriolar vasodilation and a resultant increase in renal plasma flow [19]. Initially, this translates into a compensatory rise in glomerular filtration rate; however, persistent hyperfiltration eventually drives glomerular enlargement, mesangial expansion, podocyte stress, and progressive podocyte loss [19,20]. Clinically, these histological changes manifest as proteinuria, albuminuria, and glomerulosclerosis. Increased glomerular capillary pressure appears to be a key contributor to this progressive injury, positioning hyperfiltration not merely as an early marker of SCN but as an active driver of its advancement [16,21].

#### 3.1.4. Fourth Hit: Tubular Injury

The renal tubules are highly susceptible to ischemic damage, driven by recurrent hypoxia, iron toxicity, oxidative stress, and hemoglobin-mediated toxicity [22,23]. This damage manifests primarily as impaired urinary concentrating ability, resulting from destruction of the vasa recta and disruption of the medullary osmotic gradient. Clinically, this presents as polyuria, nocturia, and an increased risk of dehydration, alongside distal tubular dysfunction that can progress to incomplete distal renal tubular acidosis, potassium abnormalities, and impaired urinary acidification [5].

#### 3.1.5. Fifth Hit: Oxidative Stress

Oxidative stress acts as a major amplifier of renal injury in SCN, arising from multiple overlapping sources—hemolysis, ischemia–reperfusion injury, activated leukocytes, free iron, and reactive oxygen species (ROS) generation. These reactive species contribute to endothelial injury, tubular apoptosis, and fibrogenesis. Critically, oxidative stress establishes a self-perpetuating cycle of renal injury that persists even between overt vaso-occlusive crises (VOCs) [16,18,22].

#### 3.1.6. Sixth Hit: Inflammation

SCN is increasingly recognized as a chronic inflammatory and vasculopathic state in which persistent activation of immune cells and proinflammatory cytokine pathways contributes to a self-amplifying cycle of renal injury. This inflammatory milieu promotes leukocyte adhesion, endothelial dysfunction, oxidative injury, and progressive glomerular and tubulointerstitial damage, ultimately contributing to fibrosis and nephron loss [22].

#### 3.1.7. Seventh Hit: Podocyte Injury

Podocytes are central to maintaining the glomerular filtration barrier. Hyperfiltration, oxidative stress, ischemia, and cytokine exposure are the main causes of podocyte injury and loss. Podocyte loss results in albuminuria, proteinuria, and focal segmental glomerulosclerosis (FSGS). FSGS is one of the hallmark glomerular lesions in SCN [19,21].

#### 3.1.8. Eighth Hit: Fibrosis and Chronic Kidney Disease Progression

Persistent injury activates the profibrotic pathways. The key mediators include transforming growth factor-β (TGF-β), connective tissue growth factor, and fibroblast activation. The histologic findings include interstitial fibrosis, tubular atrophy, glomerulosclerosis, and vascular remodeling. These changes become largely irreversible and drive CKD progression [22].

#### 3.1.9. Ninth Hit: Genetic Susceptibility

Not all patients with sickle cell disease develop severe nephropathy. This observation suggests a genetic contribution [24]. Although SCD is caused by a single pathogenic β-globin mutation, the risk and severity of kidney disease are influenced by additional genetic factors. The strongest evidence involves *APOL1*, *α*-*thalassemia*, and HMOX1. APOL1 G1/G2 high-risk genotypes have been associated with higher albuminuria, lower eGFR, faster CKD progression, and greater risk of kidney failure in SCD [25,26]. In contrast, co-inherited α-thalassemia appears to reduce hyperfiltration and albuminuria [27]. HMOX1 variants, particularly longer promoter GT repeats, have also been associated with lower eGFR and increased kidney injury, although findings are less consistent [26]. Other potential modifiers include *BCL11A*; *MYH9*; *ACKR1/Duffy*; *ACE*; and variants involved in HbF regulation, heme metabolism, inflammation, and endothelial function. Recent systematic reviews have also identified emerging associations involving genes such as *CRYL1*, *VWF*, *ADAMTS7*, and *LRP1B*, although these findings require further validation [24,27].

Higher fetal hemoglobin levels modify this risk and lead to reduced risk of sickling, hemolysis, and renal injury [26]. Table 2 summarizes different genetic modifiers in SCN and the supporting evidence level of each one:

#### 3.1.10. Tenth Hit: Acute Kidney Injury (AKI)

Episodes of AKI act as additional accelerators of chronic damage. Common triggers include severe VOC, sepsis, volume depletion, nephrotoxic medications, and rhabdomyolysis. Each AKI episode leaves residual nephron loss, increasing the risk of CKD progression [28].

**Figure 1:**
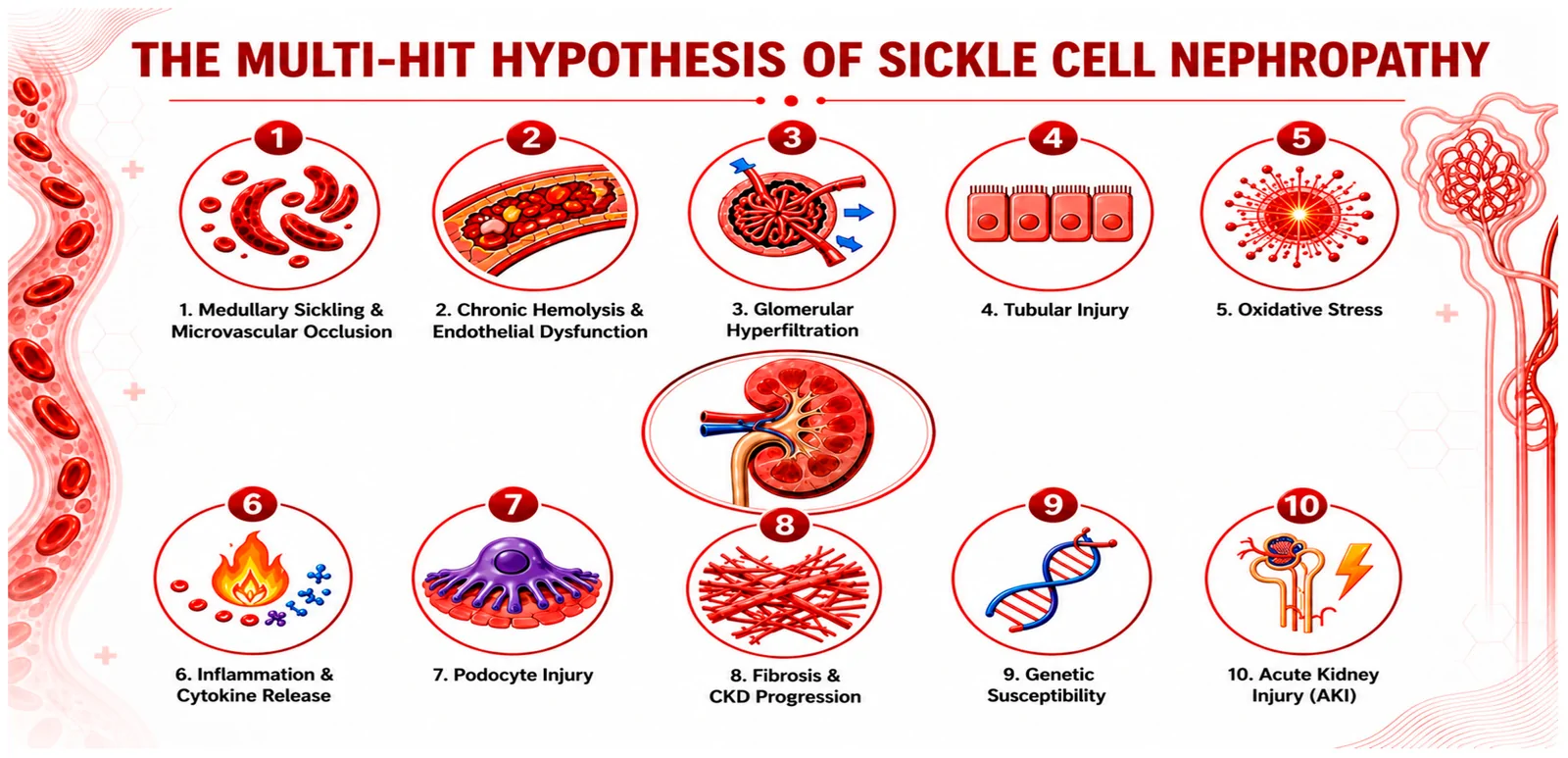
The multi-hit hypothesis of sickle cell nephropathy.

The ten interconnected mechanisms—spanning vascular, glomerular, tubular, and systemic pathways—are summarized in Figure 1, illustrating how repeated and overlapping insults converge to drive the progression from early renal dysfunction to established CKD in SCN.

### 3.2. Diagnostic Dilemmas

Diagnosing SCN remains difficult despite advances in understanding its biology. Clinical presentation is heterogeneous, biomarkers are often nonspecific, and kidney biopsy carries procedural risks that limit routine use [8]. SCN also overlaps clinically with other glomerular diseases, adding further diagnostic uncertainty. Because traditional kidney injury markers perform poorly in SCD and biopsy confirmation is rarely obtained, diagnosis is frequently delayed, with real clinical consequences given how heavily renal disease contributes to SCD morbidity and mortality [29,30]. The following sections explain why standard CKD investigations translate poorly to SCN.

#### 3.2.1. Laboratory Markers

##### Serum Creatinine

Serum creatinine underestimates kidney injury. Reduced muscle mass, increased tubular creatinine secretion, and hyperfiltration all lower serum creatinine independent of true renal function in SCD. Substantial nephron loss can occur before creatinine rises; therefore, a normal level does not exclude SCN [31].

##### Estimated Glomerular Filtration Rate (eGFR) Equations Are Inaccurate

Standard equations (CKD-EPI, MDRD, and Cockcroft–Gault) tend to overestimate renal function in SCD, risking missed early CKD and delayed treatment. This is compounded by hyperfiltration—one of the earliest signs of SCN, often defined by a measured GFR above the expected range for age and body size—which is often misread as reassuring rather than pathological [32,33].

##### Albuminuria Is Neither Sensitive nor Specific

It remains the most widely used screening marker, but false negatives occur with tubular injury or declining GFR without albuminuria, while false positives arise from fever, infection, exertion, or hypertension. Albuminuria alone cannot reliably confirm or exclude SCN [6,34].

Together, these limitations explain why SCN diagnosis is so often delayed in clinical practice.

#### 3.2.2. Histopathological Spectrum of Sickle Cell Nephropathy

Renal biopsy studies demonstrate that sickle cell nephropathy (SCN) is not a single histopathological entity but rather a heterogeneous spectrum of glomerular, tubular, vascular, and tubulointerstitial abnormalities [6]. Early renal changes include glomerular enlargement and hypertrophy, mesangial hypercellularity, congestion of glomerular capillaries, and hemosiderin deposition within tubular epithelial cells. Glomerular basement membrane thickening or reduplication and podocyte foot-process effacement have also been described. These abnormalities are thought to reflect the combined effects of chronic glomerular hyperfiltration and hypertension, medullary hypoxia and vaso-occlusion, intravascular hemolysis, and heme-mediated oxidative and endothelial injury [19,21,35].

With progression of renal disease, more advanced structural abnormalities may develop, including focal and global glomerulosclerosis, tubular atrophy, interstitial fibrosis, interstitial inflammation, and vascular injury. Tubular hemosiderosis is particularly characteristic and reflects the renal consequences of chronic intravascular hemolysis and filtered hemoglobin/heme. Medullary vascular injury and loss of the vasa recta, together with recurrent microvascular sickling, may additionally contribute to papillary and tubulointerstitial injury. Thus, the pathological phenotype of SCN reflects injury across multiple renal compartments rather than isolated glomerular disease [36,37].

Among the glomerular lesions, focal segmental glomerulosclerosis (FSGS) is the most consistently recognized pattern associated with chronic sickle cell glomerulopathy. In a series of 18 renal biopsies from patients with SCD and glomerular involvement, FSGS was identified in 39% of cases, membranoproliferative glomerulonephritis (MPGN) in 28%, thrombotic microangiopathy (TMA) in 17%, and a specific sickle cell disease-associated glomerulopathy characterized by glomerular hypertrophy and capillary congestion in 17%. Earlier biopsy studies also demonstrated marked glomerular enlargement and predominantly perihilar FSGS, supporting a relationship between chronic hyperfiltration, glomerular hypertension, and progressive glomerulosclerosis in SCD [35,36].

Age and disease stage may influence the histopathological phenotype. In a multicenter study of 36 children and adolescents with SCD undergoing kidney biopsy, mesangial hypercellularity was observed in 75% of biopsies, while FSGS, MPGN, and TMA were identified in 30%, 16%, and 2%, respectively. These findings suggest that mesangial and hyperfiltration-related abnormalities may be prominent earlier in the course of renal disease, whereas glomerulosclerosis and chronic tubulointerstitial changes may become more evident with progressive renal injury [38,39,40].

Importantly, the identification of a glomerular lesion in a patient with SCD does not necessarily establish that the lesion is attributable solely to SCD. Although FSGS and the characteristic glomerular hypertrophy of sickle cell glomerulopathy are considered closely related to the renal hemodynamic and vascular abnormalities of SCD, MPGN, TMA, immune-complex–mediated disease, and other glomerular lesions may represent superimposed or alternative renal pathology [40]. Accordingly, kidney biopsy should be considered when the clinical phenotype is atypical for conventional SCN, particularly in patients with abrupt or nephrotic-range proteinuria, nephrotic syndrome, dysmorphic or persistent hematuria, active urinary sediment, features suggestive of another systemic disease, or a rapid or otherwise unexplained decline in kidney function [3,41].

Recognizing these distinct pathological patterns is clinically important because some may represent potentially treatable coexisting kidney diseases rather than irreversible manifestations of SCN.

#### 3.2.3. Imaging Challenges

Conventional ultrasound may show enlarged kidneys and increased echogenicity, but these findings are nonspecific, while Doppler studies can assess renal blood flow and screen for renal vein thrombosis [42]. Emerging magnetic resonance imaging (MRI) techniques—including blood oxygen level-dependent (BOLD) MRI and diffusion-weighted imaging—show promise for detecting medullary hypoxia before structural injury becomes apparent [43,44].

### 3.3. Differential Diagnosis Challenges

#### 3.3.1. Differential Diagnosis

Patients with SCD may develop kidney diseases unrelated to sickling. Conditions mimicking SCN are summarized in Table 3.

Without biopsy, differentiation is often impossible. Hematuria is another diagnostic puzzle in SCN. It may result from papillary necrosis, renal medullary ischemia, Nutcracker syndrome, renal vein thrombosis, glomerulonephritis, and renal medullary carcinoma [38]. The latter is particularly concerning as it may initially present as isolated hematuria and be mistaken for benign SCN [35,37,41]. Accordingly, a stepwise approach to suspected SCN evaluation is postulated (Table 4).

#### 3.3.2. Future Directions to Alleviate Diagnostic Challenges in SCN

Future diagnostic strategies will likely integrate multi-biomarker panels, emerging biomarkers (Table 5), proteomics and metabolomics, urinary extracellular vesicles, artificial intelligence-based risk prediction, functional MRI biomarkers, and genetic risk stratification, including APOL1 status. Such approaches may enable earlier diagnosis, before irreversible fibrosis develops [45,46].

#### 3.3.3. Emerging Biomarkers

Several candidate biomarkers are under investigation to improve diagnostic accuracy, including glomerular, tubular, hemolytic, and inflammatory pathways (Table 5). Although promising, none have yet replaced albuminuria or biopsy in routine clinical practice [47,48].

### 3.4. Therapeutic Landscape and Management Strategies in Sickle Cell Nephropathy

The management of SCN aims to prevent disease progression, mitigate complications, and improve patient outcomes. Current strategies involve a combination of disease-modifying therapies, renal-protective agents, and supportive care [49].

#### 3.4.1. Disease-Modifying Therapies

##### Hydroxyurea (HU)

HU remains the cornerstone of treatment for SCD, reducing VOC and improving survival [50]. Its renoprotective effects are thought to result from increased fetal hemoglobin (HbF), which reduces HbS polymerization, red blood cell sickling, and hemolysis. In addition, its anti-inflammatory and antioxidant properties may help reduce kidney injury [51]. The evidence supporting a definitive renoprotective effect in SCN remains limited. In the BABY HUG multicenter randomized, double-blind, placebo-controlled trial, renal function was a co-primary outcome; hydroxyurea did not significantly alter measured GFR after 24 months, although it was associated with improved urine-concentrating ability and reduced renal enlargement. These findings suggest potential benefits on early renal manifestations of SCD but do not establish an effect on long-term CKD progression or kidney failure [52]. However, because 40–50% of HU is excreted by the kidneys, dose adjustment is required in patients with CKD to prevent drug accumulation and toxicity. Current recommendations generally suggest the following adjustment in patients with impaired kidney function (Table 6) [53]:

These renal dose adjustments are supported primarily by pharmacokinetic data and prescribing information rather than SCN-specific clinical trials. Hydroxyurea should therefore be individualized according to hematologic response, renal function, and treatment-related toxicity, with careful monitoring for myelosuppression [54]. The potential benefit of hydroxyurea in reducing sickling-related renal injury remains biologically plausible but should not be interpreted as definitively established renoprotection in SCN. Importantly, HU has no known direct nephrotoxic effects. Instead, by increasing HbF and reducing red blood cell sickling, it may decrease hyperfiltration, albuminuria, and recurrent ischemic kidney injury [55,56]. Therefore, the main concern in CKD is hematologic toxicity due to reduced renal clearance rather than direct kidney toxicity.

##### Chronic Red Blood Cell Transfusions

Regular red blood cell transfusions reduce HbS levels, decrease sickling, and help prevent vaso-occlusive complications. They may also provide renal benefits by reducing hemolysis and improving renal oxygen delivery [57,58]. However, repeated transfusions increase the risk of alloimmunization, which can complicate future kidney transplantation by increasing the risk of allograft rejection [59]. Therefore, chronic transfusions should be used cautiously in transplant candidates.

##### Hematopoietic Stem Cell Transplantation (HSCT) and Gene Therapy

Curative and potentially curative therapies, including hematopoietic stem cell transplantation (HSCT) and autologous gene therapy, offer the prospect of fundamentally altering the natural history of sickle cell disease by eliminating or substantially reducing the production of sickle hemoglobin. By reducing chronic hemolysis, vaso-occlusion, endothelial injury, and recurrent ischemic injury, these approaches may also prevent further renal damage and potentially modify the course of SCN [60,61]. However, their renal benefits should currently be interpreted cautiously, as neither HSCT nor gene therapy has been evaluated in randomized controlled trials with kidney-specific outcomes as primary endpoints.

HSCT is an established curative treatment for selected patients with sickle cell disease. Limited observational evidence suggests that successful transplantation may reduce glomerular hyperfiltration and stabilize renal function, although reductions in estimated GFR after transplantation may partly reflect normalization of pre-existing hyperfiltration rather than true loss of kidney function. Albuminuria may persist or transiently worsen following transplantation, and acute kidney injury related to conditioning, transplantation, or other post-transplant complications remains an important concern [62]. Thus, although HSCT may prevent ongoing sickling-mediated injury and potentially preserve renal function, definitive evidence that it reverses established SCN, reduces albuminuria, or prevents progression to kidney failure is lacking. Its broader application is further limited by donor availability and the risks associated with conditioning, graft failure, graft-versus-host disease, infertility, infections, and other transplant-related toxicities [60,63].

Gene therapy and gene-editing approaches provide an alternative strategy by modifying autologous hematopoietic stem cells, thereby avoiding the need for an allogeneic donor and the risk of graft-versus-host disease [63]. Early clinical trials have demonstrated substantial and sustained reductions in vaso-occlusive events together with improvements in hemoglobin and hemolytic parameters. These effects provide a strong biological rationale for potential long-term protection against progressive organ injury, including SCN. Nevertheless, current trials have primarily focused on hematologic and vaso-occlusive outcomes, and robust data on albuminuria, eGFR trajectories, CKD progression, or kidney failure remain unavailable. In addition, gene therapy requires stem-cell mobilization and collection, specialized manufacturing, myeloablative conditioning, and long-term follow-up, while treatment-related toxicity, fertility concerns, cost, infrastructure requirements, and limited global accessibility remain substantial barriers to widespread implementation [63,64].

Overall, HSCT currently has direct but predominantly observational evidence suggesting potential stabilization or modification of renal abnormalities in SCD, whereas the potential renoprotective effects of gene therapy remain largely hypothesis-generating. Both approaches should therefore be regarded primarily as disease-modifying or curative strategies for the underlying hematologic disorder, with possible secondary renal benefits that require confirmation in prospective studies incorporating predefined kidney-specific endpoints. Future studies should assess longitudinal changes in albuminuria, measured and estimated GFR, tubular injury biomarkers, acute kidney injury, and progression to kidney failure to determine whether correction of the sickling phenotype can truly prevent or reverse established SCN.

#### 3.4.2. Renal-Protective Agents

##### Renin–Angiotensin–Aldosterone System (RAAS) Inhibitors

Angiotensin-converting enzyme (ACE) inhibitors and angiotensin receptor blockers (ARBs) are the mainstay of treatment for proteinuria and hypertension in SCN. They reduce intraglomerular pressure, decrease proteinuria, and help slow CKD progression [65]. Although long-term SCN-specific data remain limited [66], their use is recommended in patients with proteinuria, with regular monitoring of serum creatinine, potassium, and blood pressure. Ongoing hemolysis is not a contraindication to therapy; however, treatment should be reassessed if there is a >30% rise in serum creatinine, symptomatic hypotension, hyperkalemia, or acute kidney injury [67]. In advanced CKD, continuation should be individualized, and ACEi/ARBs may still be beneficial if tolerated. In patients receiving dialysis, these agents are mainly used for hypertension, heart failure, or left ventricular hypertrophy, as their antiproteinuric effect becomes less relevant once residual kidney function is minimal [67,68].

##### Sodium–Glucose Cotransporter 2 (SGLT2) Inhibitors

SGLT2 inhibitors, originally developed for type 2 diabetes, have shown substantial renal and cardiovascular benefits in chronic kidney disease including those without diabetes. Preclinical evidence suggests potential renoprotective effects in SCD, but clinical evidence in SCN remains very limited [69]. In the DAPA-CKD trial, dapagliflozin significantly reduced the composite of sustained ≥50% eGFR decline, kidney failure, or renal/cardiovascular death, with similar benefits in participants with and without diabetes [70]. Similarly, EMPA-KIDNEY demonstrated a significant reduction in kidney disease progression or cardiovascular death with empagliflozin across a broad CKD population, including patients without diabetes [71]. During acute vaso-occlusive episodes, acute chest syndrome, sepsis, significant volume depletion, acute kidney injury, or major surgery, temporary interruption should be considered, consistent with general SGLT2 inhibitor sick-day principles. SCD-specific safety and sick-day guidance remain insufficiently studied [72].

These potential benefits are biologically relevant to SCN because glomerular hyperfiltration, intraglomerular hypertension, oxidative stress, and tubular injury are prominent components of its pathogenesis. SGLT2 inhibition reduces intraglomerular pressure through restoration of tubuloglomerular feedback and may also attenuate tubular stress and injury, providing a mechanistic rationale for investigation in SCN [11].

However, evidence supporting their use specifically in SCN remains limited. Patients with sickle cell disease were not represented in the major SGLT2 inhibitor outcome trials to an extent that permits conclusions regarding efficacy in SCN; consequently, the substantial renal benefits demonstrated in conventional CKD populations cannot be assumed to translate directly to this distinct disease setting [70,71].

##### Other Emerging Agents

Several novel therapies are being investigated for their potential renoprotective effects in SCN. Endothelin receptor antagonists (ERAs), such as atrasentan and sparsentan, have demonstrated, based on evidence from preclinical models, mechanistic rationale, and limited early clinical data, antiproteinuric and renoprotective effects in other proteinuric kidney diseases by reducing endothelin-1-mediated vasoconstriction, inflammation, and fibrosis [73]. Preliminary direct clinical evidence is also available from a small randomized phase 1 study of ambrisentan in patients with SCD, which demonstrated acceptable short-term tolerability and a signal toward reduced albuminuria. However, the study was not powered to establish renal efficacy, and clinical evidence demonstrating effects on eGFR decline, CKD progression, or kidney failure remains lacking [74]. Thus, ERAs should currently be considered an investigational, hypothesis-generating approach for SCN, rather than an established renoprotective therapy.

Similarly, pyruvate kinase activators, including Mitapivat and Etavopivat, improve red blood cell energy metabolism, reduce sickling and hemolysis. Their potential renal benefit is biologically plausible because reducing HbS polymerization and hemolysis may mitigate mechanisms implicated in SCN [75]. However, evidence specifically supporting renoprotection remains limited to exploratory clinical observations and mechanistic or preclinical data, with no adequately powered studies demonstrating reductions in albuminuria, preservation of eGFR, or prevention of CKD progression or kidney failure. Accordingly, their role in SCN should currently be regarded as hypothesis-generating and investigational, requiring prospective studies with predefined renal outcomes [75,76,77].

#### 3.4.3. Anemia Management in ESKD

##### Use of Erythropoiesis-Stimulating Agents (ESAs)

The use of erythropoiesis-stimulating agents (ESAs) in SCN requires careful patient selection, as anemia in sickle cell disease is primarily caused by chronic hemolysis rather than erythropoietin deficiency [78]. In advanced CKD, reduced erythropoietin (EPO) production further worsens anemia, and ESAs may help reduce transfusion requirements and the risk of alloimmunization. However, higher doses are often needed because of ongoing hemolysis and inflammation [79]. The American Society of Hematology (ASH) guideline conditionally suggests combining an ESA with hydroxyurea in adults and children with SCD and worsening anemia associated with CKD; however, the certainty of evidence is very low and the available studies were limited largely to HbSS and HbSβ^0^-thalassemia [61,80].

ESAs may be considered particularly when anemia is accompanied by a decline in absolute reticulocyte count despite optimized hydroxyurea therapy. Retrospective data suggest that ESA therapy does not necessarily increase vaso-occlusive crisis frequency, but evidence remains limited. Treatment should be used cautiously, with close monitoring for hypertension, thrombosis, and excessive hemoglobin rise, particularly in patients receiving hydroxyurea [79,80]. Hemoglobin targets should therefore be individualized according to symptoms, transfusion requirements, and thrombotic or vaso-occlusive risk rather than applying a validated SCN-specific target. ASH recommends avoiding hemoglobin levels > 10 g/dL (hematocrit > 30%) during ESA therapy because of concerns regarding vaso-occlusive complications, stroke, and venous thromboembolism. Thus, ESA use in SCN represents a disease-specific guideline-supported but low-certainty practice, rather than an intervention supported by high-quality randomized evidence [61,81].

##### Management of Volume in SCN with ESKD

Diuretics are the mainstay of treatment for fluid overload in CKD, but they should not be used routinely in patients with SCN and end-stage kidney disease. Their use should be guided by the patient’s volume status and residual kidney function. Excessive diuresis can cause dehydration, increase blood viscosity, promote red cell sickling, and precipitate VOC. In patients on hemodialysis, it may also increase the risk of intradialytic hypotension and ischemic complications. Careful assessment of volume status is therefore essential, as both fluid overload and dehydration are associated with adverse outcomes [82].

#### 3.4.4. Dialysis and Kidney Transplantation in ESKD

Patients with SCN who progress to ESKD require renal replacement therapy (RRT), either dialysis or kidney transplantation.

##### Dialysis

Patients with sickle cell disease have poorer outcomes on dialysis than the general dialysis population because of a higher risk of intradialytic hypotension, vascular access complications, thrombosis, and recurrent VOC [82,83]. Anemia is often more difficult to manage, frequently requiring higher doses of erythropoiesis-stimulating agents and occasional blood transfusions. Both hemodialysis (HD) and peritoneal dialysis (PD) are acceptable treatment options. HD is more commonly used but requires careful attention to volume removal and hemodynamic stability. PD offers better hemodynamic stability and may preserve residual kidney function; however, peritonitis can precipitate VOC, and protein losses may worsen malnutrition [83].

Practical considerations during hemodialysis in patients with sickle cell disease include avoiding high ultrafiltration rates and excessive interdialytic weight gain, maintaining careful assessment of volume status, avoiding unnecessary intravascular volume depletion, and closely monitoring for intradialytic hypotension and vaso-occlusive complications. During an acute vaso-occlusive episode, reduction in ultrafiltration goals may be considered, while intravenous fluid administration should be reserved for documented or clinically suspected volume depletion. Hemoglobin management should also be individualized, with avoidance of excessive correction; an SCD-specific dialysis review suggested that hemoglobin should generally not exceed approximately 10 g/dL, whereas current CKD anemia guidelines recommend an ESA maintenance target below 11.5 g/dL in the general CKD population [84].

##### Kidney Transplantation

Kidney transplantation should be strongly considered in patients with SCN who develop kidney failure and should not be withheld because of sickle cell disease alone. In a large USRDS/UNOS/OPTN analysis, transplantation was associated with a substantial reduction in mortality compared with remaining on dialysis or the transplant waiting list, with a similar relative survival benefit to that observed in patients without SCD [85]. Although contemporary transplant outcomes remain somewhat inferior to those of recipients without SCD, transplantation remains an important treatment option for appropriately selected patients [86]. In a contemporary UNOS/OPTN analysis, SCD was associated with higher mortality and lower death-censored graft survival than other causes of kidney failure; however, these findings should not discourage transplantation in appropriately selected patients [87]. Recurrence of SCN in the renal allograft is an important consideration because the underlying sickling disorder persists after transplantation. Recurrent SCN, including intragraft vaso-occlusive injury, has been reported after transplantation and may contribute to graft dysfunction or loss. In a transplant series, recurrent SCN accounted for 5 of 13 graft losses, highlighting that recurrence can be clinically significant [85,86].

Long-term graft outcomes therefore depend not only on conventional transplant factors but also on control of sickling-related complications. Post-transplant management should emphasize adequate hydration, prevention and prompt treatment of vaso-occlusive episodes and infection, and careful monitoring of graft function and proteinuria [87,88]. Strategies to reduce HbS burden, including pre- or post-transplant red-cell exchange transfusion, may be considered in selected high-risk patients. In a retrospective multicenter series, post-transplant automated exchange transfusion was associated with improved patient and graft survival and better graft function, although these findings remain observational and are potentially confounded by differences in patient characteristics and transplant era [88].

Hydroxyurea may have a role in reducing ongoing sickling-related allograft injury, but evidence in kidney transplant recipients is limited; its use requires careful monitoring for myelosuppression, particularly when combined with other myelosuppressive post-transplant medications such as mycophenolate, valganciclovir, and trimethoprim-sulfamethoxazole [84,87]. Corticosteroid exposure should be individualized, given concerns that steroid exposure may precipitate vaso-occlusive pain; nevertheless, steroid-containing immunosuppression regimens have been successfully used after kidney transplantation in patients with SCD when clinically indicated.

An important consideration is the timing of kidney transplantation and HSCT. The optimal sequencing of HSCT and kidney transplantation in SCN remains uncertain, as no comparative studies or formal guidelines address this question. When both therapies are being considered, correction of the underlying sickling disorder before or around kidney transplantation may theoretically reduce recurrent allograft injury; however, kidney transplantation should not be delayed in patients with ESKD who are not suitable candidates for immediate HSCT. Management should therefore be individualized by a multidisciplinary team involving nephrology, hematology, and transplantation teams, with consideration of disease severity, HSCT eligibility, donor availability, transplant risk, and the likelihood of recurrent sickling complications, taking into account donor availability, transplant eligibility, disease severity, and the risks of HSCT [89].

### 3.5. Limitations

As a narrative review, this study is subject to limitations inherent to the design. The literature is heterogeneous in patient age, SCD genotype, definitions of kidney disease, and outcome assessment, and much of the SCN-specific therapeutic evidence comes from small observational studies or extrapolation from general CKD populations. The search was restricted primarily to the English-language literature and was not accompanied by a formal meta-analysis or quantitative risk-of-bias synthesis. Landmark studies published before the main search period were retained when identified through reference-list screening. These limitations should be considered when interpreting treatment recommendations and the proposed research priorities.

## 4. Knowledge Gaps and Research Imperatives

Despite advances in understanding and managing SCN, substantial knowledge gaps remain. Importantly, research priorities are not uniform across settings: some are universal, whereas others are particularly relevant to resource-limited regions, where the burden of SCD is greatest but access to diagnostic and renal replacement services remains limited [81,82].

### 4.1. Universal Research Priorities

- Early diagnosis and accurate assessment of kidney function: Validated biomarkers capable of detecting early tubular and glomerular injury before irreversible damage are urgently needed. Prospective studies are also required to determine the most accurate methods for estimating GFR in SCD, including validation of cystatin C-based and combined creatinine–cystatin C equations in such populations.
- Long-term evaluation of renoprotective therapies: Robust prospective studies are needed to establish the long-term effects of RAAS blockade and to determine whether SGLT2 inhibitors and GLP-1 receptor agonists prevent CKD progression and kidney failure in SCD. Importantly, SCD-specific renal outcome data remain limited for most emerging therapies.
- Genetic and clinical risk stratification: Further research is needed to define the contribution of APOL1 and other genetic, hematologic, and clinical factors to SCN susceptibility and progression and to determine whether these markers can be incorporated into clinically useful risk-prediction models.
- Renal effects of disease-modifying SCD therapies: The long-term effects of hydroxyurea and newer therapies, including L-glutamine and crizanlizumab, on albuminuria, eGFR decline, and ESKD require systematic evaluation. These studies should incorporate renal outcomes as prespecified endpoints rather than secondary observations.

### 4.2. Priorities for High-Burden, Resource-Limited Settings

- Locally relevant epidemiological data and implementation research: Multicenter longitudinal cohorts are needed to define the incidence, natural history, and determinants of SCN in African populations, including the contribution of genetic diversity, environmental factors, recurrent dehydration, infections, and limited access to disease-modifying therapy.
- Feasible approaches to screening and diagnosis: Research should prioritize affordable and scalable strategies for SCN screening, including standardized urine albumin assessment and practical approaches to estimating kidney function where cystatin C testing, specialized biomarkers, or kidney biopsy are not readily available.
- Access to disease-modifying and kidney-protective therapies: Implementation studies are needed to identify barriers to hydroxyurea, RAAS inhibitors, SGLT2 inhibitors, and other evidence-based therapies, while evaluating cost, availability, adherence, monitoring requirements, and safety in local healthcare systems.
- Kidney replacement therapy and transplantation: Given the limited availability and high cost of dialysis and transplantation in many high-burden regions, research should evaluate strategies for earlier CKD detection and prevention of kidney failure, as well as models to improve equitable access to dialysis, transplantation, and multidisciplinary nephrology–hematology care.

Addressing these priorities will require international collaboration and research models that include populations from both high-resource and high-burden, resource-limited settings. Such efforts are essential to ensure that advances in SCN prevention and treatment are applicable across diverse populations and healthcare systems.

## 5. Conclusions

Sickle cell nephropathy remains a formidable challenge in the management of sickle cell disease. While significant progress has been made in understanding its complex pathophysiology and developing therapeutic strategies, an “unfinished story” persists. The insidious nature of renal damage, diagnostic complexities, and the need for more robust long-term efficacy data for existing and emerging therapies underscore the critical need for continued research. Future efforts must focus on developing accurate early diagnostic tools, conducting large-scale prospective trials for renal-protective agents, integrating genetic risk factors into personalized management, and addressing health disparities. Addressing these knowledge gaps will be essential to improve early detection, slow the progression of kidney disease, and ultimately improve long-term outcomes for individuals living with sickle cell nephropathy.

## Figures and Tables

**Table 1:** Inclusion and exclusion criteria.

Criteria	Inclusion	Exclusion
Study Type	Randomized controlled trials (RCTs), observational studies (cohort, case–control), systematic reviews, meta-analyses, and authoritative clinical guidelines.	Case reports (unless highlighting unique diagnostic challenges), editorials, commentaries, and conference abstracts without full-text availability.
Language	Articles published in English.	Non-English publications without available translations.
Focus	Studies specifically addressing renal manifestations of SCD, diagnostic hurdles, or therapeutic interventions.	Studies on SCD without specific renal outcomes or general CKD studies not involving SCD patients.
Population	Pediatric and adult populations with confirmed SCD genotypes.	Animal models (unless providing critical pathophysiological insights not available in human studies).

**Table 2:** Genetic modifiers in SCN and the supporting evidence level.

Genetic Modifier	Main Renal Association	Evidence
APOL1 G1/G2	↑ albuminuria, ↓ eGFR, ↑ CKD progression/ESKD	Relatively strong
HBA1/HBA2 (α-thalassemia)	↓ hyperfiltration and albuminuria	Relatively consistent
HMOX1	↓ eGFR; possible ↑ AKI risk	Inconsistent
BCL11A	Possible protection from albuminuria	Limited
MYH9	Proteinuria/renal dysfunction	Limited
ACKR1/Duffy	Possible ↑ proteinuria	Conflicting
CRYL1, VWF, ADAMTS7, LRP1B	Emerging associations with renal outcomes	Preliminary

↑: Increased, ↓: Decreased.

**Table 3:** List of SCN mimickers.

Disease	Diagnostic Challenge
FSGS	Can occur as primary disease or secondary SCN
IgA nephropathy	Hematuria overlaps with SCN
Membranous nephropathy	Heavy proteinuria
Lupus nephritis	Similar urinary findings
HIV-associated nephropathy	Common in some populations
Diabetic nephropathy	Increasing prevalence
Infection-related GN	Common in endemic regions

**Table 4:** Stepwise approach to suspected SCN evaluation.

Step	Key Actions
Step 1: Clinical assessment	Evaluate SCD genotype, frequency of VOC, history of AKI, hypertension, and family history of CKD.
Step 2: Laboratory evaluation	Urinalysis; urine albumin–creatinine ratio; serum creatinine; cystatin C; combined eGFR estimates using creatinine and cystatin C; hemolytic markers.
Step 3: Assess for alternative diagnoses	Red flags: nephrotic-range proteinuria (>3.5 g/day), rapid eGFR decline, active urinary sediment, low complement levels, positive autoimmune serology.
Step 4: Kidney biopsy	Consider when proteinuria is disproportionate, renal function declines rapidly, or an alternative glomerular disease is suspected.

**Table 5:** List of emerging biomarkers in SCN by nephron segment.

Glomerular	Tubular	Hemolysis	Inflammatory Markers
Nephrin	NGAL	Plasma free hemoglobin	TNF-α
Podocin	KIM-1	LDH	IL-6
VEGF	NAG	Haptoglobin	MCP-1

**Table 6:** Hydroxyurea dose adjustment in kidney dysfunction.

Kidney Function	Hydroxyurea Dose
Creatinine clearance ≥ 60 mL/min	Standard dose
Creatinine clearance < 60 mL/min	Start at ~50% of standard dose
Hemodialysis	Give after dialysis, usually reduced dose

## Data Availability

The Data presented in this study is derived from public domain resources and are available in the corresponding references.

## Abbreviations

**Abbreviation**	**Meaning**
SCD	Sickle cell disease
SCN	Sickle cell nephropathy
CKD	Chronic kidney disease
ESKD	End-stage kidney disease
HbS	Hemoglobin S
HbF	Fetal hemoglobin
APOL1	Apolipoprotein L1
FSGS	Focal segmental glomerulosclerosis
MPGN	Membranoproliferative glomerulonephritis
TMA	Thrombotic microangiopathy
HIV	Human immunodeficiency virus
GN	Glomerulonephritis
AKI	Acute kidney injury
eGFR	Estimated glomerular filtration rate
CKD-EPI equation	Chronic kidney disease epidemiology equation
MDRD equation	Modification of diet in renal disease equation
LDH	Lactate dehydrogenase
TNF	Tumor necrosis factor
IL-6	Interleukin-6
MCP	Monocyte chemoattractant protein-1
MRI	Magnetic resonance imaging
HU	Hydroxyurea
HSCT	Hematopoietic stem cell transplantation
ACEi	Angiotensin-converting enzyme inhibitors
ARB	Angiotensin receptor blockers
RAAS	Renin–angiotensin–aldosterone system
SGLT2	Sodium–glucose cotransporter 2 inhibitors
ERA	Endothelin-receptor antagonist
ESA	Erythropoiesis-stimulating agent
EPO	Erythropoietin
VOC	Vaso-occlusive crises
RRT	Renal replacement therapy
HD	Hemodialysis
PD	Peritoneal dialysis
GLP-1 agonists	Glucagon-like peptide 1 agonists
